# Effect of exogenous calcitriol on myopia development and axial length in guinea pigs with form deprivation myopia

**DOI:** 10.1038/s41598-024-62131-x

**Published:** 2024-05-18

**Authors:** Rongbin Liang, Wenqing Shi, Tao Li, Hui Gao, Ting Wan, Bing Li, Xiaodong Zhou

**Affiliations:** 1 Department of Ophthalmology, Jinshan Hospital of Fudan University, No. 1508 Longhang Road, Jinshan District, Shanghai, 201500 China; 2 Department of Anatomy & Embryology, Maastricht University, Maastricht, The Netherlands; 3 Department of Central Laboratory, Jinshan Hospital, Fudan University, Shanghai, China

**Keywords:** Calcitriol, Vitamin D receptor, Myopia, Choroid, Sclera, Metabolomics, Eye diseases, Molecular medicine

## Abstract

The annual increase in myopia prevalence poses a significant economic and health challenge. Our study investigated the effect of calcitriol role in myopia by inducing the condition in guinea pigs through form deprivation for four weeks. Untargeted metabolomics methods were used to analyze the differences in metabolites in the vitreous body, and the expression of vitamin D receptor (VDR) in the retina was detected. Following form deprivation, the guinea pigs received intraperitoneal injections of calcitriol at different concentrations. We assessed myopia progression using diopter measurements and biometric analysis after four weeks. Results indicated that form deprivation led to a pronounced shift towards myopia, characterized by reduced choroidal and scleral thickness, disorganized collagen fibers, and decreased scleral collagen fiber diameter. Notably, a reduction in calcitriol expression in vitreous body, diminished vitamin D and calcitriol levels in the blood, and decreased VDR protein expression in retinal tissues were observed in myopic guinea pigs. Calcitriol administration effectively slowed myopia progression, preserved choroidal and scleral thickness, and prevented the reduction of scleral collagen fiber diameter. Our findings highlight a significant decrease in calcitriol and VDR expressions in myopic guinea pigs and demonstrate that exogenous calcitriol supplementation can halt myopia development, enhancing choroidal and scleral thickness and scleral collagen fiber diameter.

## Introduction

Myopia is a common condition that primarily occurs in children and early adulthood. It is mainly characterized by excessive elongation of the eye, causing the image of distant objects to focus in front of the retina, ultimately leading to blurred distance vision^1^. Globally, myopia is recognized as a disease of high prevalence. Recent years have witnessed a yearly increase in the incidence of myopia-related diseases, including severe visual impairment and blindness^2,3^. In certain countries and regions within East and Southeast Asia, the prevalence rates of myopia among high school students soar to an astonishing 80–90%^1^. A discernible trend towards the "juvenilization" of myopia is evident, wherein early-onset myopia is more likely to advance to high myopia, markedly elevating the risk of irreversible visual impairment^4^. The pathogenesis of myopia is multifaceted and complex, with numerous underlying mechanisms of its development still shrouded in ambiguity, culminating in a conspicuous absence of effective treatments for myopia at present^5^. Consequently, the quest for effective strategies to prevent the progression of myopia assumes paramount importance.

Presently, drawing upon a comprehensive body of evidence from observational studies, randomized controlled trials, and experimental research, the principal strategies for the prevention of myopia encompass a spectrum of interventions. These include optical measures (such as frame glasses and contact lenses), environmental modifications (notably, augmenting time spent outdoors and curtailing the extent of near-vision tasks), pharmacological approaches (employing low-dose atropine), and surgical alternatives^1,5,6^. The efficacy of these intervention strategies in mitigating myopia across a span of one year ranges merely from 11.0% to 54.3%^6^. Moreover, challenges including the phenomenon of myopia rebound, adverse effects of medications, and the variability in control outcomes underscore the lack of a universally efficacious approach to the prevention and management of myopia.

Outdoor activities are universally acknowledged as the most secure method for the prevention and control of myopia^3^. Empirical evidence suggests that augmenting outdoor activity by an additional hour weekly can mitigate the risk of myopia development by 2%^7^. A contemporary randomized controlled trial has demonstrated that an increase in the duration of outdoor activities significantly reduces the incidence of myopia, particularly among non-myopic children^8^. The precise mechanisms underlying the protective effect of outdoor activities against myopia remain elusive, with current research proposing multiple theories such as the Vitamin D hypothesis, the spectral hypothesis, and the dopamine hypothesis^9^. Studies have unequivocally shown that diminished serum concentrations of 25(OH)D correlate with an elevated risk of developing myopia. The Vitamin D hypothesis suggests that augmented exposure to ultraviolet radiation facilitates the enhanced synthesis of Vitamin D, thereby playing a pivotal role in curtailing the progression of myopia. This hypothesis is based on insights from observational studies that have suggested a potential association between myopia and human vitamin D levels, but this association has not been confirmed by a large number of experimental studies. Accordingly, it is imperative to conduct experiments utilizing animal models to definitively ascertain the role of Vitamin D in the genesis and evolution of myopia.

Calcitriol, recognized as 1,25-(OH)2D3, represents the bioactive form of vitamin D, serving as a potent activator of the Vitamin D Receptor (VDR)^10^. It fulfills an indispensable role in facilitating the absorption of calcium within the small intestine and orchestrating the regulation of inorganic salt transport within the skeletal structure^11^. In the course of recent studys, calcitriol has been identified to exert substantial influence on cellular proliferation, differentiation, and the modulation of immune responses^12–14^. Calcitriol is currently being researched for its potential use in the treatment of diseases such as cancer^15^, heart disease^16^ and periodontitis^17^. Considering the obscure mechanisms underlying the involvement of vitamin D and the VDR in myopia development, it is posited that calcitriol occupies a pivotal role in the genesis of myopia. To illuminate this hypothesis, a form deprivation myopia (FDM) model in guinea pigs were be established, aimed at investigating the alterations in the expression of calcitriol, vitamin D, and VDR within FDM group. Subsequently, by administering different concentrations of calcitriol via intraperitoneal injection, the effects of calcitriol on the refractive status, axial length, choroid, and sclera structure of FDM animals will be explored.

Our study revealed that in FDM guinea pigs, there was a notable escalation in refractive error and axial length, accompanied by a significant reduction in the thickness of both choroidal and scleral tissues. The concentrations of calcitriol, vitamin D, and VDR were significantly lower in the FDM guinea pigs. The application of calcitriol at varying concentrations effectively suppressed the progression of refractive error toward myopia and the elongation of axial length in FDM animals. This treatment significantly ameliorated the thinning of both guinea pig choroid and scleral tissues while inhibiting the reduction in scleral collagen diameter.

## Materials and methods

### Animals

In accordance with the ARVO guidelines for the ethical use of animals in ophthalmic and vision research, all animals utilized in this study were appropriately housed and cared for. All animal-related procedures received approval from the Ethics Committee of Jinshan Hospital affiliated with Fudan University. Male tricolor guinea pigs aged 2 weeks and weighing 120 ± 10 g (Changyi Experimental Animal Breeding Co., Ltd., Danyang City, Jiangsu, China) were maintained in a controlled environment with a 12-h light (300 lx) and 12-h dark cycle, at an ambient temperature of approximately 22 ± 2 °C, with access to ample and consistent supplies of water and food daily. Prior to the start of the experiment, guinea pigs presenting with antimetropia (> 2 diopter (D)), congenital myopia, or other ocular abnormalities (such as lens opacities or congenital developmental anomalies) were excluded from the study.

All experiments in this study were approved by the Ethics Committee of Jinshan Hospital, Fudan University (JIEC2023-S92). All experimental procedures and related operations in this study were carried out in accordance with the standards of ARRIVE guidelines (PLoS Bio 8(6), e1000412,2010). All Animals were anesthetized and sacrificed according to the American Veterinary Medical Association (AVMA) Guidelines for the management of Animals (2020).

### Induction of form deprivation myopia

Monocular deprivation was induced to create FDM using a method of covering one eye^18^. In brief, a translucent milky-white balloon was fashioned into a head cover to occlude the right eye, while ensuring the left eye, both ears, and the nose and mouth were fully exposed and free to move. This arrangement allowed the guinea pigs to eat, breathe, and turn their heads without restriction. The right eye was covered from baseline (0 week, 0W). The fit of the head cover was checked every day at 8 a.m. and 4 p.m., and any displacement or detachment was immediately adjusted and re-applied. The head cover was worn continuously until the end of the experiment.

### Refractive error measurements

Refractive measurements for both eyes of all animals were taken at 0W, after 2 weeks (2W), and after 4 weeks (4W) using a small animal photorefractor (SriaTech Company, Germany) set at the following parameters: wavelength: 875 nm; frame rate: > 115 Hz; working distance: 56 cm. Before each measurement, pupils were dilated using a compound tropicamide eye drop (Santen Pharmaceutical Co., Ltd., Japan) for 10 min. All measurements were conducted in a dark room, and the average value of the refractive error was recorded and saved once it stabilized.

### Axial length measurements

Using an animal A-scan ultrasound device (OD1-A, Kexin Electronic Equipment Co., Ltd., Xuzhou, China), with the guinea pig measurement mode selected, axial lengths of both eyes were measured for all animals at 0W, and then at 2W and 4W post-covering. Prior to measurement, tetracaine hydrochloride eye drops (Santen Pharmaceutical Co., Ltd., Japan) were applied to the ocular surface for anesthesia. A 10 Hz ultrasound probe was vertically aligned and gently applied to the cornea, and axial length readings were obtained by the software of the device. The probe underwent disinfection following each use to ensure sterility before measurements on the subsequent eye. Data were stabilized, and the mean of three consecutive measurements was documented as the definitive result, with a precision of 0.01 mm.

### Tissue preparation

After 2W or 4W of rearing, control group guinea pigs (n = 16) and FDM group guinea pigs (n = 16) were euthanized immediately after being administered 1% pentobarbital sodium via intraperitoneal injection, and their eyeballs were removed. Under microscopic guidance, the eyeballs were promptly dissected to isolate the vitreous body and retinal tissues. Subsequently, these tissues were immediately transferred into cryotubes and submerged in liquid nitrogen for a duration of 10 min. Additionally, a volume of 3 ml of blood was collected using a disposable blood collection tube and subjected to the same freezing process. Samples for vitreous body metabonomics, blood Enzyme-linked immunosorbent Assays (ELISA), and Western Blot analysis of retinal tissue were transferred from liquid nitrogen to a storage environment of − 80 °C until required for further experiments.

### Vitreous body untargeted metabolomics analysis

A 150 μL extract solution (ACN: Methanol = 1:4, V/V) containing internal standard was added into 50 μL vitreous body. Then the sample was vortex for 3 min and centrifuged at 12,000 *rpm* for 10 min (4 °C). Take that the supernatant fluid − 20 °C placed in 3 min, and then centrifuged at 12,000 RPM for 3 min (4° C). 120ul of the supernatant was used for Liquid Chromatograph (LC-30A, Shimadzu, Japan) Mass Spectrometer(TripleTOF 6600 +, SCIEX, Foster City, CA, USA) (LC–MS) analysis.

All samples were for two LC/MS methods. One alipuot was analyzed using positive ion conditions andwas eluted from T3 column (Waters ACQUITY Premier HSS T3 Column 1.8 μm, 2.1 mm *100 mm) using 0.1% formic acid in water as solvent A and 0.1% formic acid in acetonitrile as solvent B in the following gradient:5 to 20% in 2 min, increased to 60% in the following 3 min, increased to 99% in 1 min and held for 1.5 min, then come back to 5% mobile phase B witnin 0.1 min, held for 2.4 min. The analytical conditions were asfollows, column temperature, 40 °C; flow rate, 0.4 mL/min; injection volume, 4 μL; Another alipuot was usingnegative ion conditions and was the same as the elution gradient of positive mode.

Data acquisition was conducted using the information-dependent acquisition mode, facilitated by AnalystTF 1.7.1 software (Sciex, Concord, ON, Canada). The source parameters were configured as follows: Ion Source Gas 1 at 50 psi, Ion Source Gas 2 at 50 psi, Curtain Gas at 25 psi, temperature set at 550 °C, Declustering Potential at 60 V for positive mode and − 60 V for negative mode, and Ion Spray Voltage Floating at 5000 V for positive mode and − 4000 V for negative mode. Time-of-Flight MS scan parameters included a mass range of 50–1000 Da, an accumulation time of 200 ms, and dynamic background subtraction enabled. Product ion scan parameters were set with a mass range of 25–1000 Da, an accumulation time of 40 ms, a collision energy of 30 V in positive mode and − 30 V in negative mode, a collision energy spread of 15, resolution set to UNIT, a charge state from 1, an intensity threshold of 100 cps, isotopic exclusion within a 4 Da range, a mass tolerance of 50 ppm, and a cap of 18 candidate ions monitored per cycle.

Peak extraction, peak alignment and retention time correction were respectively performed by XCMS program. Unsupervised PCA (principal component analysis) was performed by statistics function prcomp within R (www.r-project.org). The data was unit variance scaled before unsupervised PCA. The HCA (hierarchical cluster analysis) results of samples and metabolites were presented as heatmaps. For two-group analysis, differential metabolites were determined by VIP (VIP > 1) and *P*-value (*P*-value < 0.05, Student’s t test). Identified metabolites were annotated using KEGG Compound database (http://www.kegg.jp/kegg/compound/), annotated metabolites were then mapped to KEGG Pathway database (http://www.kegg.jp/kegg/pathway.html).

### Drug treatments

Calcitriol (T6316, TagetMol, USA) was initially solubilized in ethanol (100,092,683, Sinopharm Chemical Reagent Co., Ltd., China) and then diluted in 0.9% saline (Qidu Pharmaceutical Co., LTD., China) before being used in the experiments. In this study, 100 guinea pigs were randomly allocated into five groups: Control (n = 20), solvent (FDM + 0.9% NaCl, n = 20), low-dose (FDM + 0.05 µg/kg Calcitriol, n = 20), medium-dose (FDM + 0.25 µg/kg Calcitriol, n = 20), and high-dose (FDM + 0.5 µg/kg Calcitriol, n = 20). The control group did not receive any intervention. The low-dose, medium-dose, and high-dose group were given three different concentrations of pre-prepared calcitriol solutions. Based on the body weight of the guinea pigs, the appropriate volume of the medicated solution was drawn to achieve the final doses of 0.05 µg/kg, 0.25 µg/kg, and 0.5 µg/kg, respectively. Injections were administered once daily at 8 a.m. by the same qualified physician. The injection regimen started on the day of model creation and continued for 4 weeks until the end of the experiment. Using a 31G disposable micro syringe (U-100, Puang Medical Technology Co., Ltd., Hangzhou, China). All procedures were conducted under sterile conditions to mitigate the risk of infection.

### Enzyme-linked immunosorbent assays

Using ELISA kits (Enzyme-linked Biotechnology Co., Shanghai, China), the collected blood samples were quantitatively analyzed for vitamin D and calcitriol content. In accordance with the kit instructions, the supernatant was obtained for analysis following centrifugation of the blood samples. Reaction wells were populated with 50 µl of both standard and test samples. Subsequently, 50 µl of biotin-labeled antibody was introduced to each well and allowed to incubate at ambient temperature for one hour. Post three washes of the plate, 80 µl of avidin-HRP conjugate was dispensed into each well, followed by mixing and a further 30-min incubation at room temperature. After another trio of washes, 50 µl of Substrates A and B were added to the wells, mixed, and incubated in darkness at room temperature for 10 min. Upon rapid retrieval of the microplate, 50 µl of stop solution was applied, and the optical density (OD) values were measured at 450 nm wavelength using a ELISA reader (Rayto, RT-6100, China). The concentrations of vitamin D and calcitriol within the samples were subsequently quantified using a standard curve derived from the standard samples.

### Hematoxylin–Eosin (H&E) staining

Following euthanasia utilizing the previously described method, selected guinea pigs intended for pathological sectioning had their entire eyeballs immersed in a specialized fixative solution (G1109, Servicebio, Wuhan, China) and were subsequently fixed at a temperature of 4 °C for a duration of 24 h. The tissues of the eyeball were then encapsulated in paraffin for preservation. Thin sections with a thickness of 4 µm were prepared using a precision Leica microtome (RM2016, Leica, Germany). These sections underwent a series of preparatory steps including deparaffinization, staining, dehydration, and the application of a cover slip, prior to examination under a high-resolution biological microscope (E100, NIKON, Japan). The analysis and measurement of the sections were facilitated by the advanced SlideViewer software (3DHISTECH Ltd, Hungary). Measurements of the choroid and sclera thickness were specifically focused within a 1000 µm to 2000 µm range adjacent to the optic nerve.

### Immunofluorescence staining

After guinea pigs were euthanized and their tissues embedded in paraffin blocks, the sections underwent deparaffinization, antigen retrieval, and were then blocked with 10% goat serum at room temperature for 30 min. They were incubated overnight at 4 °C with the primary antibody (VDR, 67,192, Proteintech, Chicago, USA), followed by incubation with Alexa Fluor 488-labelled secondary antibody (Life Technologies, USA) for 1 h at room temperature. DAPI (D1714-QB04; Southern Biotech, China) was used for nuclear staining. Images were captured and saved using a laser microscope (TCS SP5, Leica Microsystems, Wetzlar, Germany).

### Western blot analysis

Western Blot analysis was performed on retinal tissue from the control and FDM group after 4 weeks of the experiment. Proteins from retinal tissue were extracted employing a solution that included protease and phosphatase inhibitors, along with RIPA lysis buffer (Beyotime, Shanghai, China). The protein concentrations were quantified utilizing the BCA Protein Assay Kit (Beyotime Biotechnology, China). Subsequently, uniform quantities of the freshly isolated retinal total protein (20 µg per lane) underwent electrophoresis in 10% SDS-PAGE gels, followed by their transfer onto polyvinylidene fluoride (PVDF) membranes (Millipore, IPVH00010, Germany). These membranes were then blocked for 1 h at ambient temperature in a blocking solution of 20 mM tris–HCl, 137 mM NaCl, 0.1% Tween-20 (TBST), and 5% non-fat milk. Overnight incubation at 4 °C was conducted with the primary antibody (VDR, 1:2000, 67,192, Proteintech, Chicago, USA). Following several TBST washes the next day, the membranes were incubated with a Multi-rAb HRP-Goat Anti-Mouse Recombinant Secondary Antibody (H + L) (1:3000, RGAM001, Proteintech, Chicago, USA) at ambient temperature for 1 h. Imaging of the membranes was performed using a Tanon imaging system (Shanghai, China), and the results were analyzed using Image J software (National Institutes of Health, MD, USA).

### Scanning electron microscope

Upon the euthanasia of the animals and the subsequent isolation of the cornea, lens, and vitreous body, scleral tissue samples, each with an area not surpassing 3 mm^2^ and situated proximal to the optic nerve, were meticulously collected using microscissors. The surface designated for scanning electron microscopy was carefully marked and then rapidly submerged in a 2.5% glutaraldehyde solution at ambient temperature for two hours, followed by a transfer to a 4 °C environment for preservation. Post-fixation, the specimens underwent rinsing before being subjected to a secondary fixation in a darkened setting at room temperature with 1% osmium tetroxide (18,456, Ted Pella Inc, USA) for one to two hours. Subsequent to an additional rinse, the specimens were dehydrated, air-dried, and rendered conductive. The scleral architecture was then meticulously examined and documented using a scanning electron microscope (SU8100, Hitachi, Japan) for detailed analysis.

### Transmission electron microscopy

Adhering to the established protocol for scanning electron microscopy specimen preparation, the samples, post-fixation in 1% osmium tetroxide under dark conditions at ambient temperature, underwent dehydration before being encapsulated in pure 812 resin (90529-77-4, SPI, USA), which was then introduced into embedding molds. These molds, containing the samples, were incubated in an oven set to 37 °C throughout the night. Subsequent polymerization occurred in a 60 °C oven over a span of 48 h, following which the solidified resin blocks were extracted and reserved for further processing. Ultrathin sections ranging from 60 to 80 nm were precisely sliced with an ultramicrotome (PT-PC, RMC, Germany) and mounted on 150 mesh copper grids. These grids underwent staining in a 2% uranyl acetate ethanol-based solution in a darkened environment for 8 min, followed by a triadic sequence of washes with 70% ethanol and ultrapure water. Subsequently, a 2.6% lead citrate staining, meticulous to avoid carbon dioxide exposure, was conducted for 8 min, succeeded by another series of ultrapure water rinses and gentle drying with filter paper. The prepared sections were left to air-dry at room temperature within a grid box overnight. The structural examination and imagery capture were facilitated using a transmission electron microscope (HT7800, Hitachi, Japan), with the results being subject to analytical review.

### Statistical analysis

Data are reported as the mean ± standard error of the mean. Statistical analysis and graphical representation were executed utilizing SPSS 25.0 (Chicago, IL, USA) and Prism 9 (GraphPad Software, USA). Intra-animal ocular differences were assessed using paired t-tests, whereas inter-animal ocular differences were evaluated through unpaired t-tests. Analysis across various groups was performed via one-way analysis of variance (ANOVA), followed by post hoc assessments employing either the Least Significant Difference test or Tamhane's T2 test. A *P*-value < 0.05 was deemed to indicate statistical significance.

### Ethics approval and consent to participate

This study was approved by the Ethical Committee of Jinshan Hospital of Fudan University.

## Result

### Form deprivation induces shifts in refractive error towards myopia, accompanied by alterations in the thickness of the choroid and sclera

**Figure 1:**
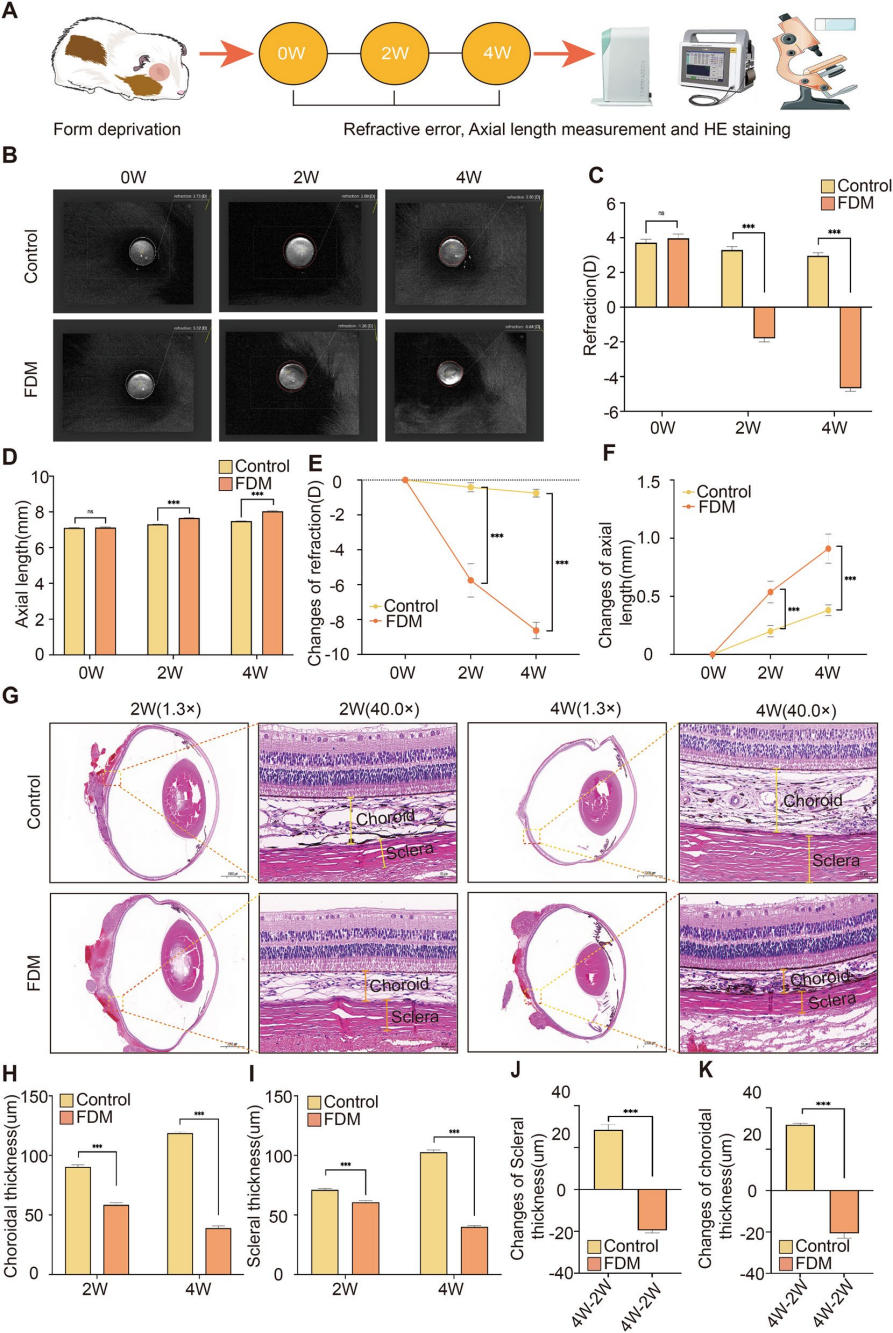
Form deprivation induces a refractive shift towards myopia, accompanied by alterations in choroidal and scleral thicknesses. (**A**) Schematic diagram of an experimental model of form deprivation myopia (FDM). (**B**) Refractive measurements at different time points. (**C**) Quantitative comparison of dioptersbetween Control group (n = 6) and FDM group (n = 6). (**D**) Comparison of axial length between FDM group and Control group at different time points. (**E**) Line plot of refractive changes. (**F**) Line chart of axial length changes. (**G**) Eyeball HE staining (1.3 × magnification (scale: 2000 μm) and detailed magnification (40.0 × , scale: 50 μm) were performed on FDM group (n = 5) and Control group (n = 5) at different time points. (**H**) Comparison of choroidal thickness at different time points. (**I**) Comparison of scleral thickness at different time points. (**J**) Changes in choroidal thickness over time. (**K**) Changes in scleral thickness over time. Data are presented as the mean ± SD. Independent t-tests comparing the two groups represented statistically significant differences. *P < 0.05, **P < 0.01, ***P < 0.001.

Measurements of refractive error, axial length, and histological analyses of the eyeball were conducted at 0, 2, and 4 weeks following form deprivation, as depicted in Fig. 1A. Initially, at baseline, the ocular biometric parameters did not significantly differ between the FDM group and the Control group, as shown in Fig. 1B–D (p > 0.05). However, after 2 and 4 weeks of form deprivation, eyes subjected to form deprivation in the FDM group exhibited a marked shift towards myopia, evidenced by significant changes in refractive error (Fig. 1B,C, p < 0.001), an increase in axial length (Fig. 1D, p < 0.001), and notable reductions in the thickness of both the choroid and sclera (Fig 1G–I, p < 0.001). Moreover, as the duration of form deprivation increased, the alterations in refractive error, axial length, and the thicknesses of the choroid and sclera became increasingly significant (Fig. 1E,F,J,K, p < 0.001).

### Vitreous body untargeted metabolomics sequencing analysis

**Figure 2:**
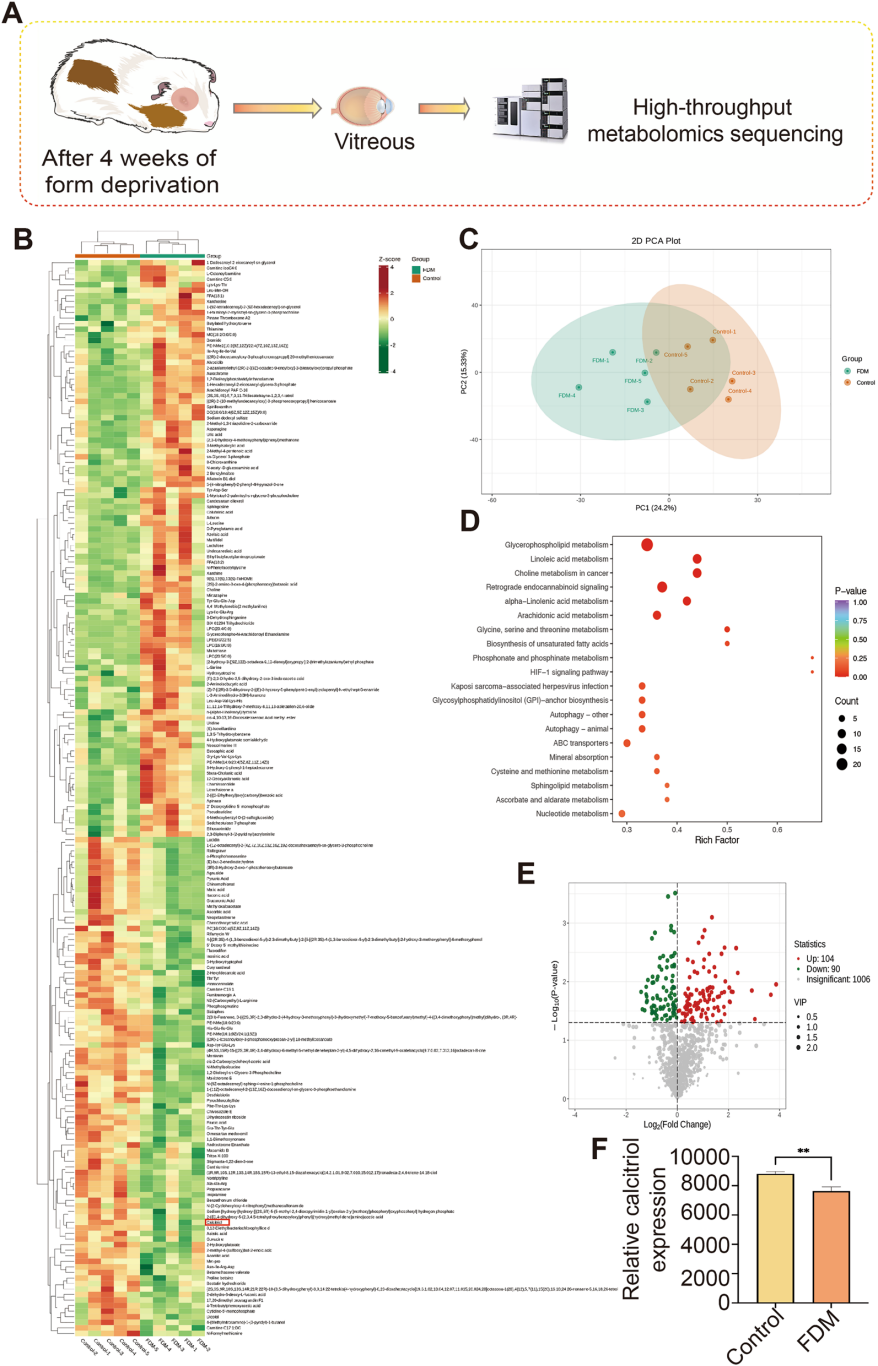
Reduction of calcitriol levels in the vitreous body of form deprivation myopia (FDM) guinea pigs. (**A**) Schematic representation of untargeted metabolomics analysis in the vitreous body of FDM guinea pig. (**B**) For all differentially expressed metabolites compared to controls, calcitriol is clearly labeled in the red box. (**C**) Principal component analysis (PCA) delineates the metabolite variability within and across the FDM group and Control group. (**D**) An enrichment analysis graph displays the top 20 enriched pathways, ordered by ascending *P*-value. (**E**) A comparison of the FDM (n = 5) and control (n = 5) groups uncovers 104 metabolites with an increased relative abundance and 90 with a decreased relative abundance. (**F**) Relative expression levels of calcitriol in the FDM and control groups, **P < 0.01. Independent t-tests comparing the two groups represented statistically significant differences. *P < 0.05, **P < 0.01, ***P < 0.001.

Following a 4-week period of form deprivation, an untargeted metabolomic sequencing analysis was performed on the vitreous body, as depicted in Fig. 2A. Prior to the differential analysis, principal component analysis (PCA) was employed to evaluate the variability amongst and within the groups under study, facilitating a differential comparison as illustrated in Fig. 2C. This analysis succeeded in identifying a total of 1,200 distinct metabolites. In comparison with the control group (n = 5), the FDM group (n = 5) exhibited an increase in 104 metabolites and a decrease in 90 metabolites in terms of relative abundance. Figure 2B and E display cluster heatmaps and volcano plots, respectively, showcasing all metabolites exhibiting differential abundance. Notably, the relative expression of calcitriol was significantly reduced in the FDM group (Fig. 2B,F, p < 0.01). Further, a differential metabolite Kyoto Encyclopedia of Genes and Genomes (KEGG) enrichment analysis, shown in Fig. 2D, revealed significant enrichment in pathways including glycerophospholipid metabolism, linoleic acid metabolism, and mineral absorption.

### Form deprivation myopia results in a diminished expression of the retinal vitamin D receptor

**Figure 3:**
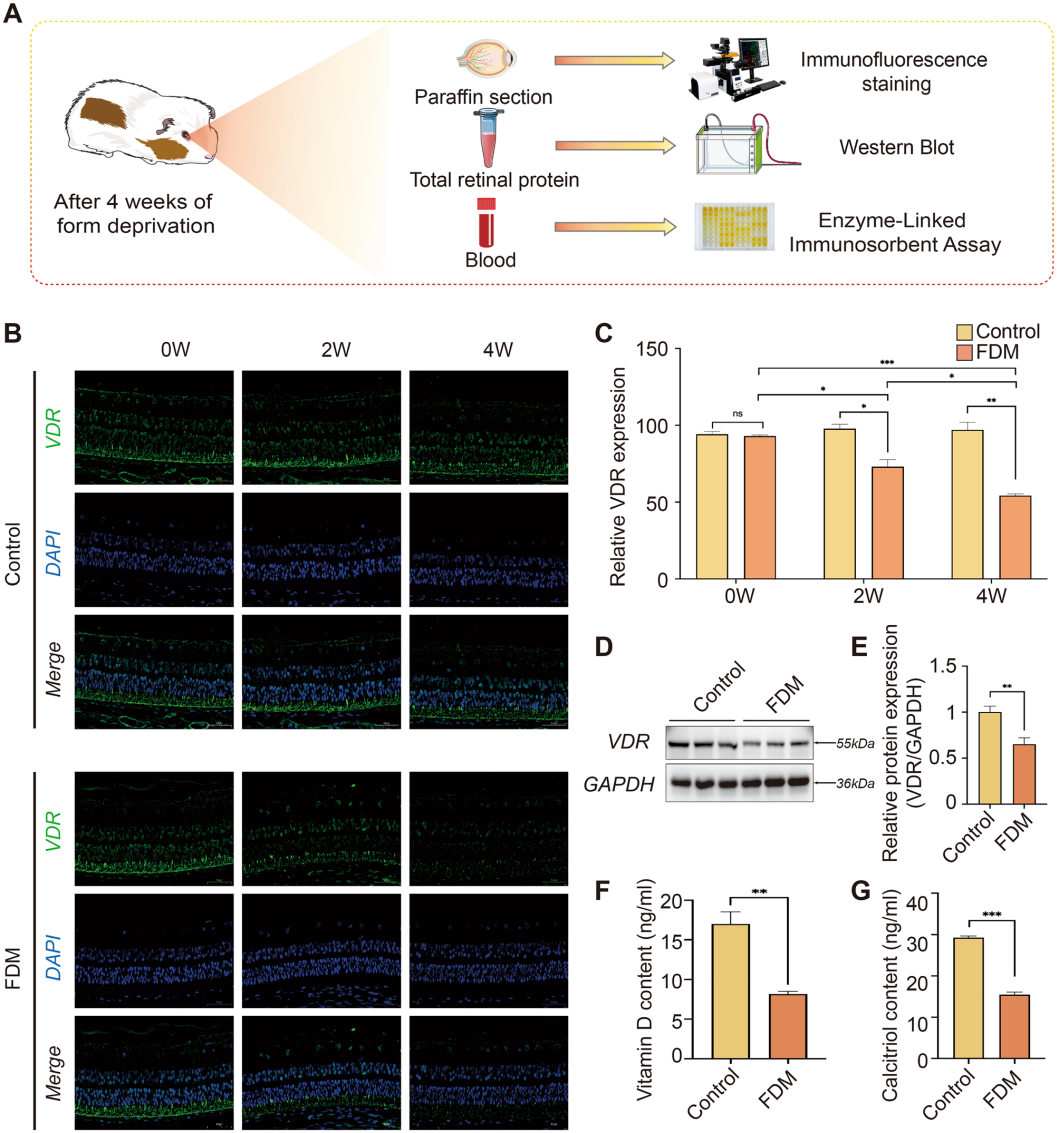
Reduced expression of calcitriol and the vitamin D receptor (VDR) in guinea pigs with form deprivation myopia (FDM). (**A**) Schematic representation of the detection of VDR in the retina and calcitriol and vitamin D in blood. (**B**) Immunofluorescence assays illustrating VDR expression in the retina at 0, 2, and 4 weeks in both the control group (n = 3) and the FDM group (n = 3). DAPI staining is shown in blue and VDR in green (scale: 50 μm). (**C**) A semi-quantitative analysis contrasts the average grayscale values of VDR expression over time between the groups, the expression of VDR decreased with time, *P* < 0.05. (**D**) Western Blot analysis assessing VDR protein levels in the retina of control (n = 6) and FDM (n = 6) groups. (**E**) Relative quantitative analysis of VDR protein expression in the retina, P < 0.001. (**F**) The serum levels of vitamin D in the control group (n = 4) and the FDM group (n = 4) were detected by ELISA, P < 0.01. (**G**) ELISA was used to detect the expression level of calcitriol in the blood of the control group (n = 4) and the FDM group (n = 4), *P* < 0.001. Independent t-tests comparing the two groups represented statistically significant differences.*P < 0.05, **P < 0.01, ***P < 0.001.

At 0, 2, and 4 weeks of form deprivation, immunofluorescence staining and Western blot analyses were conducted on ocular fundus tissues (Fig. 3A). The immunofluorescence findings indicated that the VDR was present across various retinal layers and choroidal tissues. As form deprivation was extended, the relative expression levels of VDR in the retinal tissues of the FDM group (n=3) showed a progressive decline when compared to the Control group (n=3) (Fig. 3B,C). Western blot analysis at the conclusion of the study demonstrated that VDR protein expression in the retinal tissues of the FDM group was substantially lower than in the Control group (Fig. 3D,E, p<0.001). The original images of blots for Fig. 3D are in the Supplementary Information.

### Vitamin D and calcitriol levels in the blood were reduced in form deprivation myopia in guinea pigs

Upon concluding the 4-week study and subsequent euthanasia of the guinea pigs, we utilized ELISA kits to assess the blood levels of vitamin D and calcitriol (Fig. 3A). The analysis showed that after 4 weeks, both vitamin D (Fig. 3F, p < 0.01) and calcitriol (Fig. 3G, p < 0.001) concentrations in blood were significantly lower in the FDM group compared with the control group.

### Calcitriol can significantly improve the progression of form deprivation myopia

**Figure 4:**
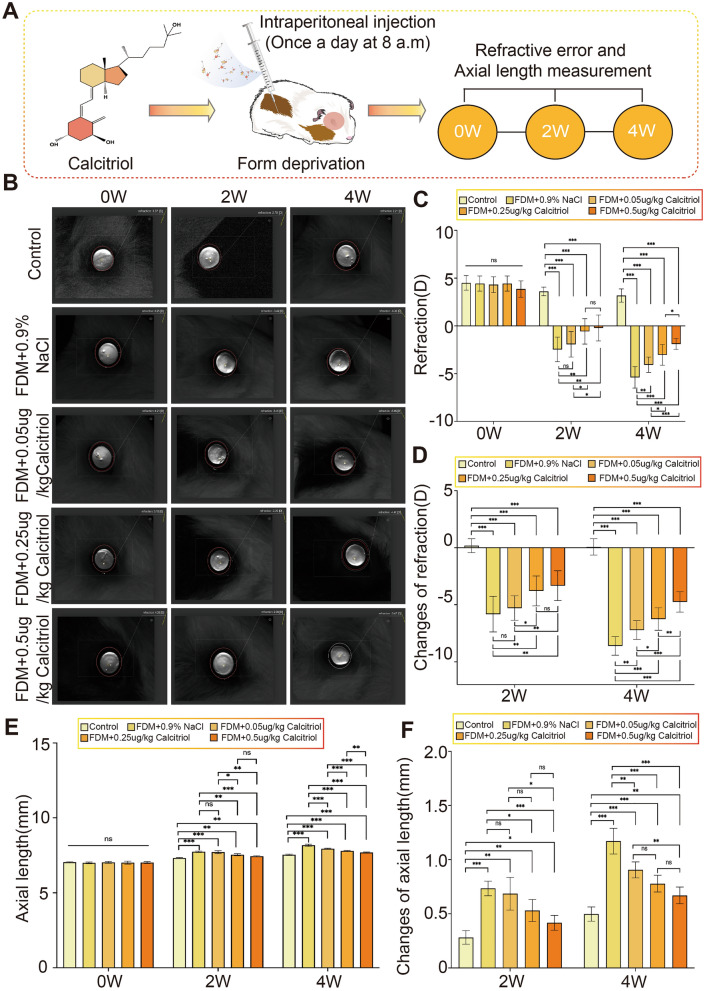
Calcitriol significantly mitigates refractive error and axial length elongation in guinea pigs with form deprivation myopia (FDM). (**A**) Schematic illustration detailing the protocol for intraperitoneal administration of calcitriol and subsequent measurement of various ocular biological parameters in FDM guinea pigs. At different time points after calcitriol treatment, (**B**) diopter measurement, (**C**) quantitative comparison of diopter, and (**D**) comparison of diopter changes. After calcitriol treatment, (**E**) quantitative comparison of axial length and (**F**) comparison of changes in axial length at different time points (n = 7 per group). Statistical analysis was performed using one-way analysis of variance (ANOVA) among multiple groups. *P < 0.05, **P < 0.01, ***P < 0.001.

Following daily intraperitoneal injections of calcitriol, we assessed refractive errors and axial lengths at 0, 2, and 4 weeks of treatment (Fig. 4A). Initially, at 0 weeks, no significant differences in refractive errors or axial lengths were detected among the control group (n = 7), the FDM + 0.9% NaCl group (n = 7), and the various calcitriol-treated groups (FDM + 0.05 μg/kg, FDM + 0.25 μg/kg, and FDM + 0.5 μg/kg, each n = 7) (Fig. 4B,C,E). After two weeks of form deprivation, all treated groups exhibited a myopic shift in refractive error and axial length growth to varying degrees compared to the control group (Fig. 4D,F). Specifically, no significant difference was found between the FDM + 0.9% NaCl group and the FDM + 0.05 μg/kg Calcitriol group, while notable differences were observed between the FDM + 0.9% NaCl group and both higher-dose calcitriol groups (FDM + 0.25 μg/kg and FDM + 0.5 μg/kg); significant distinctions were also evident between the lowest dose calcitriol group and the two higher-dose groups, with no marked difference between the two highest-dose groups. By 4 weeks, all treated groups demonstrated more pronounced myopic shifts compared to the control group (Fig. 4D,F), with significant distinctions among all calcitriol-treated groups (Fig. 4C,E). Collectively, the findings at 2 and 4 weeks suggest that calcitriol administration, relative to its absence, contributes to the mitigation of refractive error and axial length progression.

### Calcitriol can inhibit the thinning of the choroid and sclera in guinea pigs with form deprivation myopia

**Figure 5:**
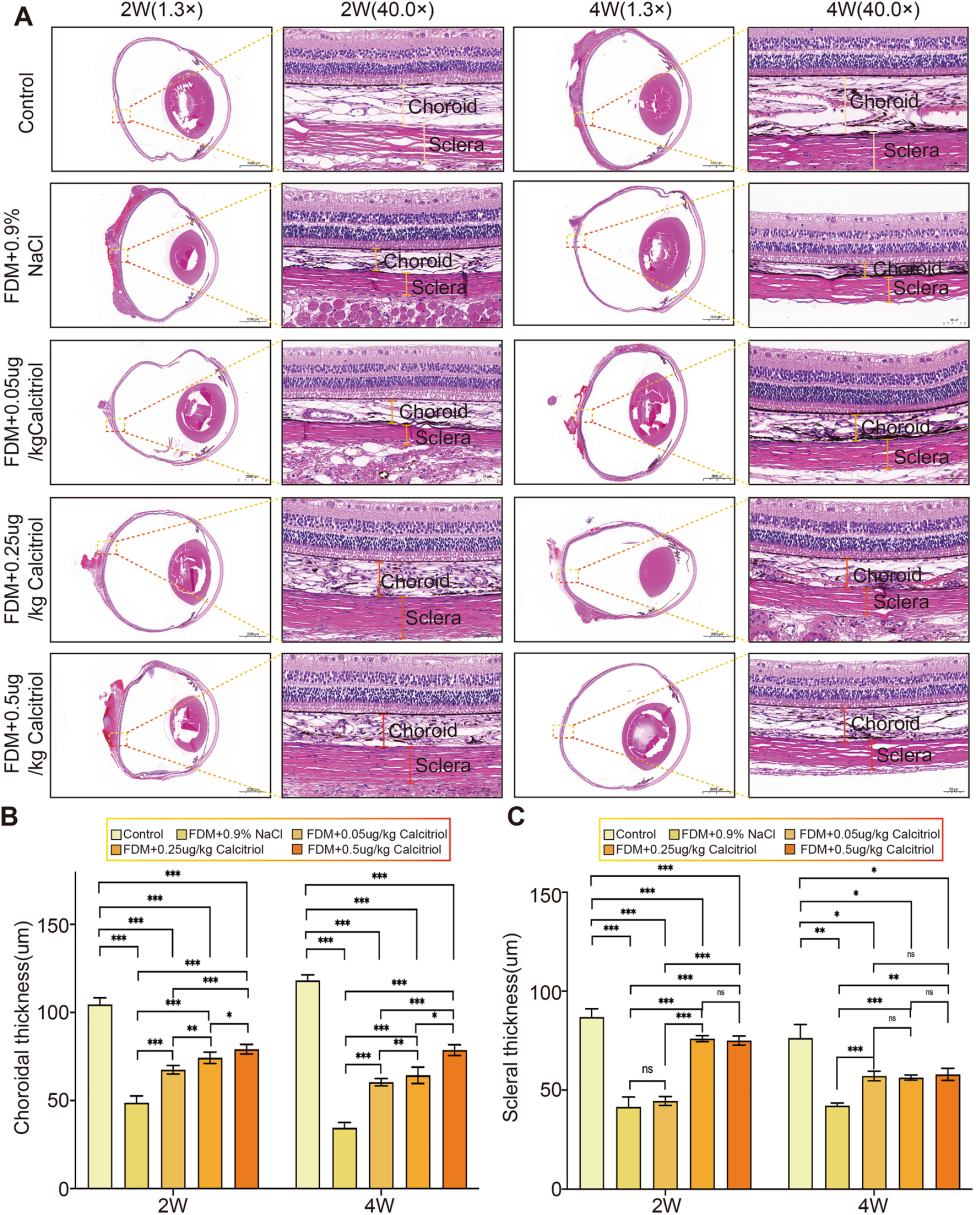
Calcitriol significantly mitigates choroid and sclera thinning in guinea pigs with form deprivation myopia (FDM). (**A**) After calcitriol treatment, Eyeball HE staining (1.3 × magnification (scale: 2000 μm) and detailed magnification (40.0 × , scale: 50 μm) were performed on FDM group (n = 5) and Control group (n = 5) at different time points. After calcitriol treatment, comparison of (**B**) choroidal thickness and (**C**) scleral thickness at different time points. Statistical analysis was performed using one-way analysis of variance (ANOVA) among multiple groups. *P < 0.05, **P < 0.01, ***P < 0.001.

Following 2 and 4 weeks of form deprivation, HE staining analyses were performed. Subsequent to form deprivation at these intervals, both choroidal and scleral thicknesses exhibited significant reductions in the FDM + 0.9% NaCl group (n = 3), as well as in the FDM + 0.05 μg/kg, FDM + 0.25 μg/kg, and FDM + 0.5 μg/kg Calcitriol groups (n = 3 in each group), when compared to the control group (n = 3) (Fig. 5A). By week 4, the extent of choroidal and scleral thinning was markedly mitigated in the Calcitriol-treated groups relative to the FDM + 0.9% NaCl group (Fig. 5B,C). Across both 2 and 4 weeks, significant variations in choroidal thickness were noted among all groups. However, at the 2-week, no significant variances in scleral thickness were observed between the FDM + 0.9% NaCl and FDM + 0.05 μg/kg Calcitriol groups, nor between the FDM + 0.25 μg/kg and FDM + 0.5 μg/kg Calcitriol groups. By week 4, similar observations of non-significant differences in scleral thickness were made between the lower and higher Calcitriol dosage groups. Collectively, the findings indicate that Calcitriol alleviated the reduction in choroidal and scleral thickness observed at 2 and 4 weeks after form deprivation.

### Exogenous calcitriol supplementation markedly ameliorates the diminished vitamin D levels in guinea pigs subjected to form deprivation myopia

**Figure 6:**
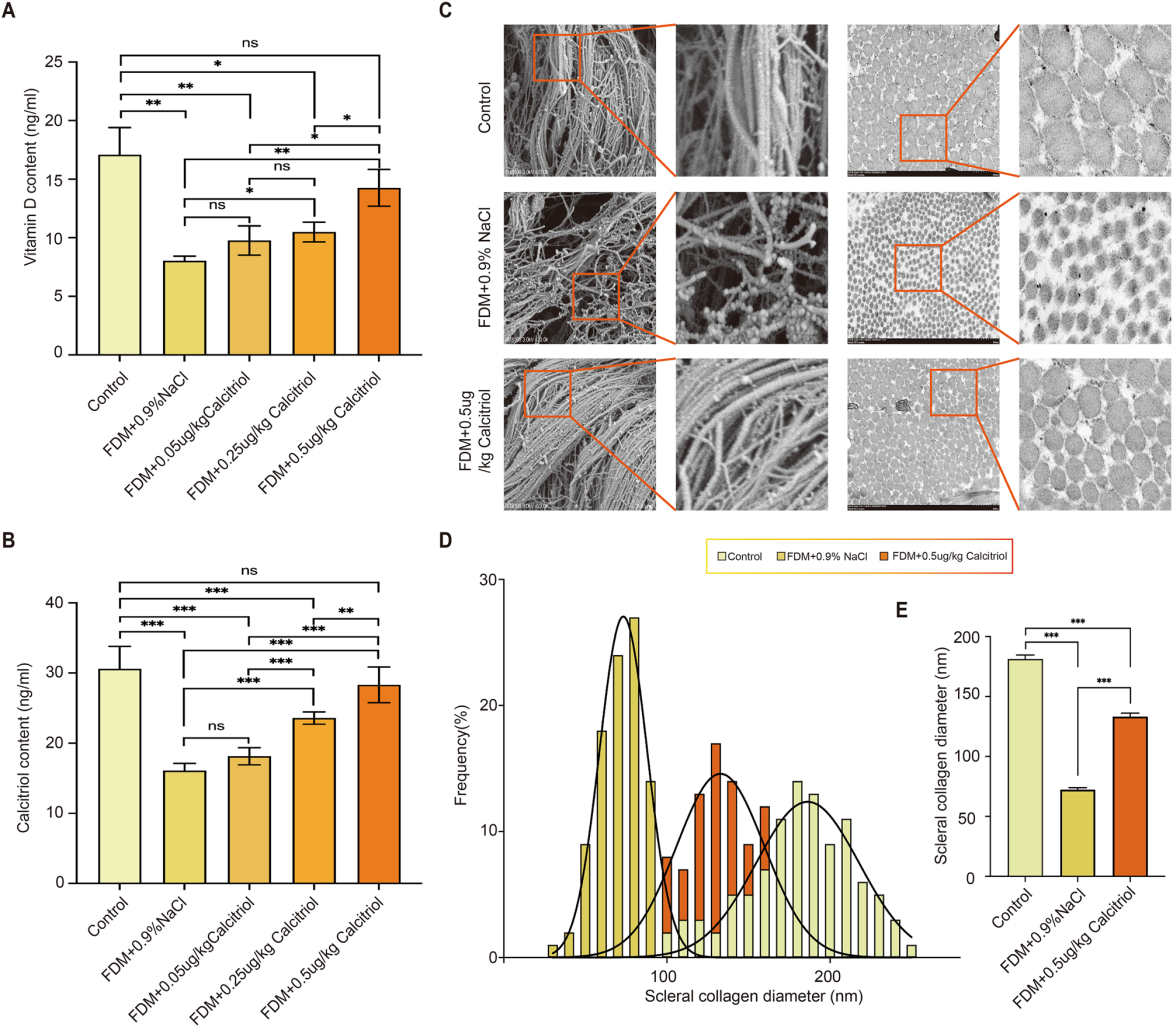
Calcitriol increase the blood levels of vitamin D and calcitriol, significantly enhance the diameter and length of scleral collagen fibers, and improve the arrangement of collagen. After calcitriol treatment, the blood concentrations of (**A**) vitamin D (n = 5 in each group) and (**B**) calcitriol (n = 5 in each group) were measured by ELISA. (**C**) Scanning electron microscopy (SEM, scale: 2 µm) and transmission electron microscopy (TEM, scale: 500 nm) were used to observe the changes of scleral surface morphology and scleral collagen diameter (n = 3 in each group). (**D**) Distribution of scleral collagen diameter and length among different groups and (**F**) comparison of scleral collagen diameter (n = 100 in each group), *P* < 0.001. Statistical analysis was performed using one-way analysis of variance (ANOVA) among multiple groups. *P < 0.05, **P < 0.01, ***P < 0.001.

After the conclusion of the experiments involving five groups of guinea pigs, we employed ELISA kits to measure the levels of vitamin D and calcitriol in their blood. The findings indicated a notable enhancement in the vitamin D levels that were diminished due to form deprivation, following the administration of exogenous calcitriol (Fig. 6A,B). This enhancement in vitamin D levels was progressively more significant with higher doses of calcitriol. Notably, it was at the dosage of 0.5 μg/kg of calcitriol that the vitamin D and calcitriol concentrations in the treatment group aligned with those observed in the control group (Fig. 6A,B).

### Exogenous calcitriol supplementation significantly enhances the morphology and diameter of scleral collagen fibers in guinea pigs with form deprivation myopia

The external morphology and collagen diameter of the sclera were analyzed by scanning electron microscopy and transmission electron microscopy, respectively. Compared with the control group, the scleral collagen of guinea pigs in the FDM group was disorganized, with reduced density and diameter. Notably, calcitriol supplementation markedly ameliorated these form deprivation myopia-induced structural alterations. After a treatment duration of four weeks, scleral collagen demonstrated improved organization, enhanced density, and increased diameter, indicating significant structural recovery (Fig. 6C–E).

## Discussion

Given the rising incidence of myopia, which now stands as a principal contributor to visual impairment globally, the quest for efficacious myopia control methods remains critical^19^. Although low-dose atropine has shown effectiveness, its application is limited by adverse effects and a rebound effect post-discontinuation^20,21^. Consequently, identifying new, safer, and more efficacious strategies for myopia management is crucial. Our study confirmed the role of calcitriol and VDR in the onset and progression of myopia. We have established that exogenous calcitriol supplementation notably curbs myopia progression and alleviates reductions in choroidal and scleral thickness as well as scleral diameter, these findings provide new insights into the development of new drugs for myopia.

The findings of this study identified a reduction in calcitriol and VDR levels during the progression of myopia. This was validated through tests conducted on multiple sites including the vitreous body, blood, and retina. Prior studys have not conclusively established a link between VDR and myopia. Research into VDR single nucleotide polymorphisms among Caucasians has unveiled correlations with mild to moderate myopia^22^. Moreover, a meta-analysis highlighted an association between reduced 25(OH)D levels and increased myopia risk^9^, while other studies contest a direct causal link between VDR-associated genes and myopia^23^, often centering on vitamin D3 blood levels. In our study, we conducted non-targeted metabolomic sequencing of vitreous body from guinea pigs of form deprivation, uncovering a marked reduction in calcitriol expression alongside persistent metabolic pathways linked to mineral absorption. Notably, we also detected decreased vitamin D and calcitriol levels in the blood, coupled with a significant downregulation of VDR protein in the retina. Combining these observations with existing clinical data, we believe that lower levels of calcitriol may play a role in the potential development of myopia by diminishing downstream biological functions and accelerating the progression of myopia.

Building on previous research^24^, our study on the effect of calcitriol on myopia in FDM guinea pigs showed that calcitriol alleviated the refractive myopic drift, axial elongation, choroidal and scleral thinning, and scleral collagen diameter reduction caused by form deprivation myopia. Further analysis showed that administering calcitriol at three distinct concentrations effectively curbed the onset of myopic refractive error and axial elongation in a dose-responsive manner. Notably, no discernible differences in the control of refractive error between the low and high concentration groups were observed until the 4W, underscoring the necessity for prolonged treatment to achieve discernible benefits from calcitriol supplementation. To validate the elevation of vitamin D post-supplementation, we measured vitamin D and calcitriol levels in the blood, confirming an increase in vitamin D content. The main cause of axial elongation is scleral collagen degradation^25^. Calcitriol can alleviate scleral collagen degradation and change the arrangement of scleral collagen. We speculate that calcitriol may mediate this effect through VDR^26^. Our findings affirm that calcitriol supplementation not only halts myopia progression but also contributes to the thickening of the choroid and sclera, along with an increase in scleral collagen diameter, providing substantial evidence for the development and application of myopia-preventative pharmaceuticals.

Further study is needed to elucidate the mechanism through which calcitriol mitigates myopia. Nevertheless, existing literature posits that vitamin D3 might modulate collagen I expression through its interaction with Transforming Growth Factor Beta(TGF-β) pathways, thus preventing the diminution of scleral collagen^27–29^. Recognized for its immunomodulatory and anti-inflammatory properties, calcitriol has been extensively applied in treating diverse conditions^12,30,31^. The link between inflammation and the development of myopia has recently received increasing attention, and many studies have shown that inflammation plays an important role in the occurrence and progression of myopia^32^. Anti-inflammatory agents like lactoferrin, diacerein, and resveratrol have been shown to thwart myopia progression by targeting retinal Mitogen-Activated Protein Kinase(MAPK) or Nuclear Factor kappa-B(NF-kB) signaling pathways^33^. Therefore, we propose that calcitriol may arrest myopia progression through the suppression of ocular inflammatory markers, a hypothesis that warrants further empirical study. Recent studies have reported alternative metabolic pathways of vitamin D mediated by cytochrome P450 side-chain cleavage enzyme CYP11A1^34^. CYP11A1 initiates hydroxylation at C-22 and C-20 of cholesterol to produce pregnenolone^35,36^. Among the various hydroxylated metabolites catalyzed by CYP11A1, 20(OH)D and its hydroxylated derivatives exhibit antiproliferative, differentiating, and anti-inflammatory effects in skin cells, comparable or superior to those of calcitriol^37,38^. In addition to the VDR, novel receptors for 20(OH)D and 20,23(OH)2D include human aryl hydrocarbon receptor (AhR), liver X receptor (LXR), and the retinoic acid receptor-related orphan receptors α and γ (RORα and RORγ) within the nuclear receptor family^39^. RORs are implicated in regulating immune and metabolic processes in various conditions such as cancer, autoimmune diseases, and metabolic disorders^40^. Thus, the physiological roles of vitamin D in the body are attributed not only to the calcitriol-VDR pathway but also to the pathways involving CYP11A1-derived vitamin D metabolites interacting with VDR or RORα/γ^41^. In this study, we primarily consider the regulatory role of calcitriol through the VDR pathway in the development and progression of myopia. However, alternative pathways and additional receptors beyond VDR are also critical considerations for our ongoing research into further mechanistic studies.

This study marks the first to report a reduction in calcitriol levels in animals with experimentally induced myopia. In addition, calcitriol supplementation can alleviate the progression of myopia and inhibit the thinning of choroid and sclera. The incidence and progression of myopia appear intricately linked to calcitriol concentrations within the body. While the precise mechanisms through which calcitriol influences myopia remain to be fully deciphered, our findings illuminate new avenues for the exploration and development of innovative myopia treatments and prevention strategies. Future research should delve into the biosynthesis and metabolic pathways of calcitriol, enhancing our comprehension of its potential roles in myopia pathogenesis and prophylaxis.

### Supplementary Information

Supplementary Figure S1.

## Data Availability

All data generated or analysed during this study are included in this published article.
