# Supplementary material for: Effect of exogenous calcitriol on myopia development and axial length in guinea pigs with form deprivation myopia
Source: Sci Rep. 2024 May 18;14:11382. doi: 10.1038/s41598-024-62131-x (PMC11102427; doi:10.1038/s41598-024-62131-x)

Supplementary Material

Effect of exogenous calcitriol on myopia development and axial length in guinea pigs with form deprivation myopia

Running head: Calcitriol for Myopia

Rongbin Liang[1],Wenqing Shi [1],Tao Li [1],Hui Gao[2],Ting Wan [1],Bing Li[3],Xiaodong Zhou [1#]

[1]Department of Ophthalmology, Jinshan Hospital of Fudan University, Shanghai, China;

[2]Department of Anatomy & Embryology, Maastricht University, Maastricht, The Netherlands

[3]Department of Central Laboratory, Jinshan Hospital, Fudan University, Shanghai, China

# Corresponding author

Xiaodong Zhou, Department of Ophthalmology, Jinshan Hospital of Fudan University, No. 1508 Longhang Road, Jinshan District, Shanghai 201508, P.R. China.

Email: xdzhou_2013@163.com.

Figure S1.The original images of blots for Figure 3D---VDR.

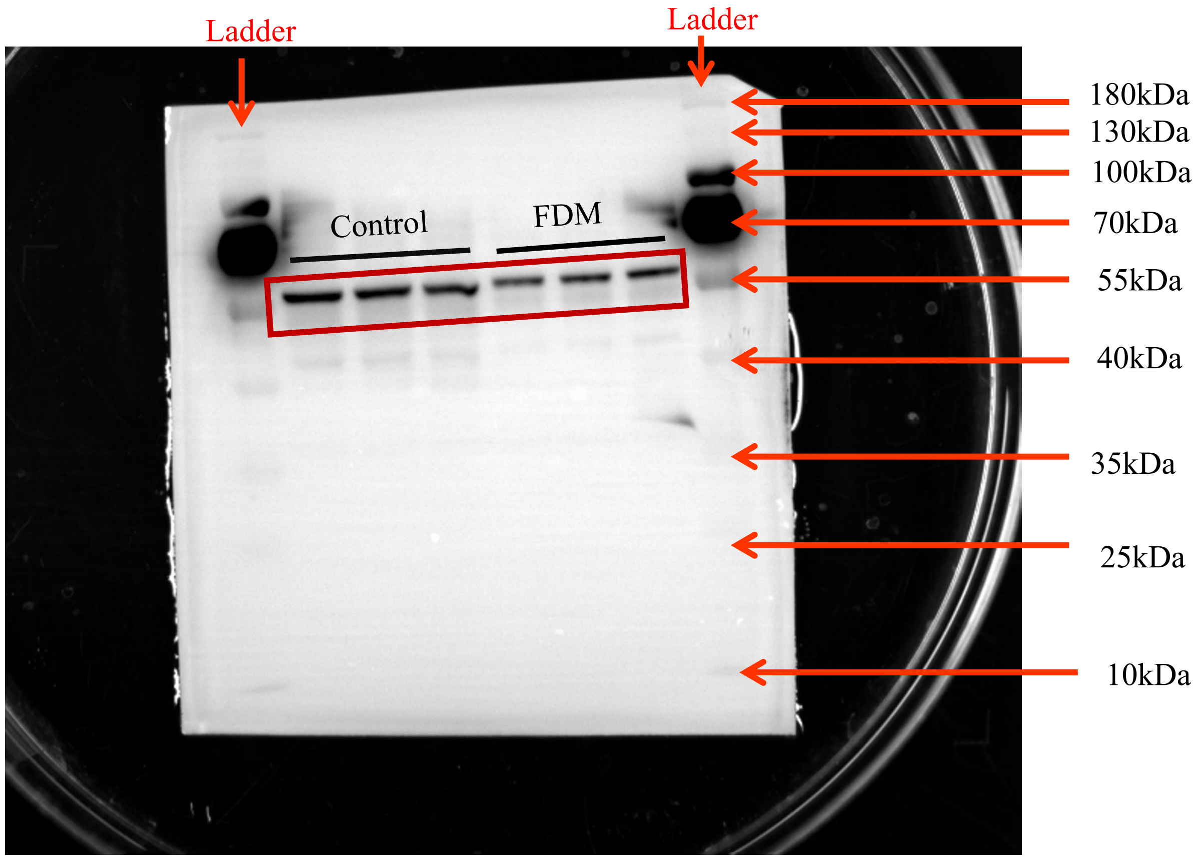

Figure S2.The original images of blots for Figure 3D---GAPDH.

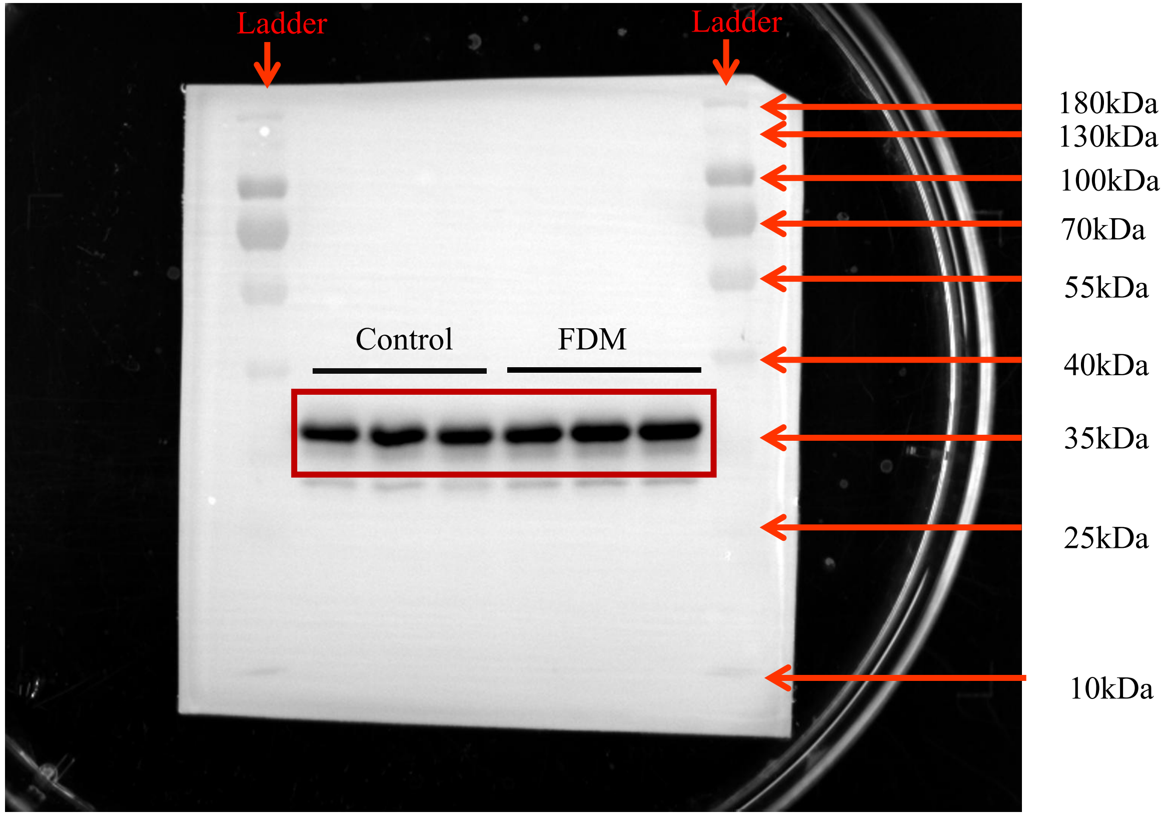

Supplement: Supplementary file 1 — Supplementary Figure S1. [file 41598_2024_62131_MOESM1_ESM.pdf]
